# Spectral Reflectance Recovery from the Quadcolor Camera Signals Using the Interpolation and Weighted Principal Component Analysis Methods

**DOI:** 10.3390/s22166288

**Published:** 2022-08-21

**Authors:** Yu-Che Wen, Senfar Wen, Long Hsu, Sien Chi

**Affiliations:** 1 Department of Electrophysics, National Yang Ming Chiao Tung University, No. 1001 University Road, Hsinchu 30010, Taiwan; 2 Department of Electrical Engineering, Yuan Ze University, No. 135 Yuan-Tung Road, Taoyuan 320, Taiwan; 3 Department of Photonics, National Yang Ming Chiao Tung University, No. 1001 University Road, Hsinchu 30010, Taiwan

**Keywords:** spectrum reconstruction, spectral reflectance recovery, linear interpolation, weighted principal component analysis, multispectral imaging, quadcolor camera

## Abstract

The recovery of surface spectral reflectance using the quadcolor camera was numerically studied. Assume that the RGB channels of the quadcolor camera are the same as the Nikon D5100 tricolor camera. The spectral sensitivity of the fourth signal channel was tailored using a color filter. Munsell color chips were used as reflective surfaces. When the interpolation method or the weighted principal component analysis (wPCA) method is used to reconstruct spectra, using the quadcolor camera can effectively reduce the mean spectral error of the test samples compared to using the tricolor camera. Except for computation time, the interpolation method outperforms the wPCA method in spectrum reconstruction. A long-pass optical filter can be applied to the fourth channel for reducing the mean spectral error. A short-pass optical filter can be applied to the fourth channel for reducing the mean color difference, but the mean spectral error will be larger. Due to the small color difference, the quadcolor camera using an optimized short-pass filter may be suitable as an imaging colorimeter. It was found that an empirical design rule to keep the color difference small is to reduce the error in fitting the color-matching functions using the camera spectral sensitivity functions.

## 1. Introduction

Multispectral imaging is an important application in color science and technology [1,2,3,4,5,6,7,8,9,10,11,12]. Spatial and spectral resolution can be improved using multiple cameras [13,14,15]. Surface spectral reflectance can be recovered from multispectral image data and the light source spectrum. Spectral reflectance can be measured directly with an imaging spectrometer, but the measurement is expensive due to the need for a diffractive optical imaging system [16,17,18]. Therefore, indirect measurements using the spectrum reconstruction technique are of interest [19,20,21,22,23,24,25,26,27,28,29]. The spectra of the image pixels are reconstructed from the channel outputs of the image acquisition device, which is an extension of the conventional trichromatic system, where the number of channels is provided by different filters, greater than three. Orthogonal projection [19], Gaussian mixture [20], principal component analysis (PCA) [21,22], non-negative matrix transformation (NMT) [23,24] and interpolation [25,26,27,28,29] have been proposed for spectrum reconstruction.

Indirect methods that require training spectra are also known as learning-based methods, such as PCA and NMT. The training spectra are used to derive basis spectra. The reconstructed spectrum is the linear combination of basis spectra. The coefficients of basis spectra can be solved from simultaneous equations describing the channel outputs of the imaging device. However, for case where XYZ tristimulus values or RGB signal values are available, only three basis spectra can be used, limiting the accuracy of the reconstructed spectra. Improved methods have been proposed to enhance the contribution of neighboring training samples of the test sample in a color space to basis spectra, such as weighted PCA (wPCA) and adaptive NMT methods [22,24]. Since basis spectra depend on the sample to be reconstructed, the computation time is significantly increased compared to conventional methods [27,29].

Another disadvantage of learning-based methods is that the equations describing the channel outputs require the spectral sensitivity functions of the imaging device for solving the coefficients of the linear combination of basis spectra. The spectral sensitivities can be directly measured using a monochromator [30], but accurate measurements are expensive. Without the use of a monochromator, the spectral sensitivities can be estimated by solving a quadric minimization problem [30,31,32]. An alternative approach is to estimate the spectral sensitivities including the device and light source so that the spectral reflectance can be calculated from signals [33,34,35,36,37,38]. The estimation errors of spectral sensitivity cause additional errors in the reconstructed spectrum.

The interpolation method uses reference spectra to reconstruct the spectrum from input values, e.g., XYZ tristimulus values [25,26,27] and RGB signal values [28,29]. Due to the use of a look-up table (LUT) to store the reference spectra, this method is often referred to as the LUT method. Since the reconstructed spectrum is interpolated from reference spectra, the LUT method does not require the spectral sensitivity functions. Furthermore, the authors of [25] showed that the LUT method has the advantage of being more accurate than the PCA method, where the reference spectra for interpolation are the same as the training spectra for the PCA method. The authors of [26,27,28,29] showed that the spectrum reconstruction errors using the wPCA and adaptive NMT methods may not be less than the LUT method. The results are reasonable because reference samples are the neighbors of the sample to be interpolated in the color or signal space.

The authors of [28,29] investigated the use of a tricolor camera for spectral reflectance recovery using the LUT method. Although tricolor cameras have only three available channels, they have the advantages of low cost and fast detection in addition to no need to measure/estimate the camera spectral sensitivity functions. The use of cameras enables more field applications, e.g., smartphone cameras used as sensors. However, if a sample is outside the convex hull of the reference samples in the signal space, it cannot be interpolated and must be extrapolated instead. The sample corresponding to such a signal vector is called an outside sample to distinguish it from the samples inside the convex hull [26,27,28,29]. The authors of [29] proposed the auxiliary reference samples (ARSs) to extrapolate the outside samples. The results showed that the extrapolation error utilizing the ARSs is lower than other methods in [27,28].

**Figure 1:**
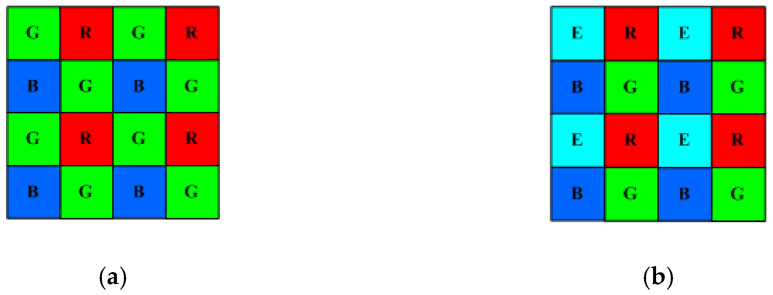
Schematic diagrams showing the (**a**) RGB color filter array and (**b**) RGBE color filter array.

This paper studies the recovery of spectral reflectance using quadcolor cameras, where four channel signals can be used to reduce spectrum reconstruction errors. The wPCA and LUT methods were used to reconstruct spectra, respectively. To the best of the authors’ knowledge, this paper is the first research work to study the use of a quadcolor camera and the LUT/wPCA method to recover surface spectral reflectance. The color filter array (CFA) on a conventional camera is shown in Figure 1a, which is known as a Bayer filter. One unit cell of the CFA includes one red, two green and one blue square filters. Each color filter corresponds to one pixel. Assuming that the irradiance varies smoothly around the unit cell, the demosaicing algorithm can be used to interpolate the missing signal of a pixel from neighboring pixels. The spatial resolution of captured images can be improved using the CFA. There are other color filter layouts, such as the RGBE filter used in the SONY Cyber-shot DSC-F828, where a green filter on the CFA unit cell is modified to a cyan filter, as shown in Figure 1b.

Due to the higher dimensional signal space, the extrapolation problem of quadcolor cameras is more severe than that of tricolor cameras using the LUT method. This paper will show that this is also a problem using the wPCA method. ARSs were used for extrapolation using the LUT method. A Nikon D5100 camera was taken as a reference tricolor camera. The RGB channels of the quadcolor cameras under consideration were assumed to be the same as the D5100 camera. The spectral sensitivity of the fourth channel was tailored using a color filter. The reflection spectra from the Munsell color chips irradiated with the illuminant D65 were taken as samples for testing.

This paper is organized as follows. Section 2.1, Section 2.2 and Section 2.3 describe the considered camera spectral sensitivities, color samples, and the assessment metrics for the recovered spectral reflectance, respectively. Four spectral sensitivity types for the fourth channel of the quadcolor camera are described in Section 2.1. Section 3.1 and Section 3.2 describe the wPCA and LUT methods, respectively. Section 3 presents a method for preparing the ARSs for extrapolation of outside samples using the quadcolor camera and the LUT method. Section 3.4 defines the factor that can be used to design the spectral sensitivities of a camera to achieve the small color difference of the reconstructed spectrum. Section 4 shows the results. Compared to the D5100 camera, the reduction in spectral reconstruction errors using the quadcolor cameras is presented. The performances using the wPCA and LUT methods are compared. The optimal designs of the considered quadcolor cameras are shown. The spectral sensitivity characteristics affecting spectral reflectance recovery were investigated. Section 5 gives the conclusions. Appendix A and Appendix B give the proofs of the zero-color-difference condition using the LUT and wPCA methods, respectively. For ease of reference, section Abbreviations lists the abbreviations defined herein in alphabetical order.

## 2. Materials and Assessment Metrics

### 2.1. Camera Spectral Sensitivities

A spectrum can be represented by the vector ***S*** = [*S*(*λ*_1_), *S*(*λ*_2_), …, *S*(*λ_M_*_w_) ]^T^, where *S*(*λ_j_*) is the spectral amplitude at wavelength *λ_j_*; *λ_j_* = *λ*_1_ + (*j* − 1)Δ*λ* is the *j*-th sampling wavelength, *j* = 1, 2, …, *M*_w_, and Δ*λ* is the wavelength sampling interval; *M*_w_ is the number of sampling wavelengths; the subscript T denotes the transpose operation. In this paper, spectra were sampled from 400 to 700 nm in a step of 10 nm, i.e., *λ*_1_ = 400 nm, Δ*λ* = 10 nm and *M*_w_ = 31. The spectral sensitivity vector of a camera signal channel can be written as

(1) $$S_{\mathrm{Cam}}=T_{\mathrm{Opt}}\circ T_{\mathrm{IRC}}\circ T_{\mathrm{CF}}\circ D,$$

where ***T***_Opt_, ***T***_IRC_ and ***T***_CF_ are the spectral transmittance vectors of the imaging lens set, IR cut filter and color filter, respectively; ***D*** is the spectral sensitivity vector of the CMOS sensor at the focal plane; and the operator ∘ is the Hadamard product, which is also known as the element-wise product. The infrared cut filter blocks the invisible infrared light, which can be replaced with the UV/IR cut filter. For simplicity, the lens transmittance was not considered in this paper.

**Figure 2:**
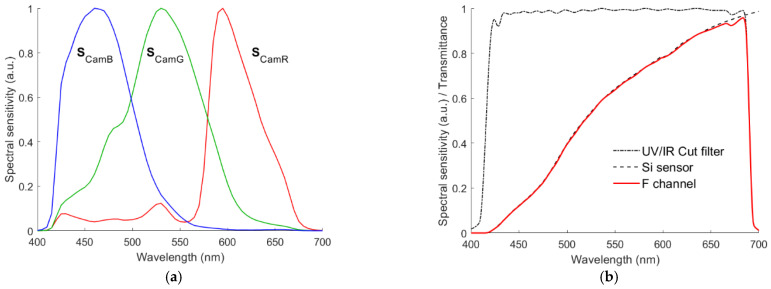
(**a**) Spectral sensitivities of the Nikon D5100 camera, where the spectra ***S***_CamR_, ***S***_CamG_ and ***S***_CamB_ are the sensitivities of the red, green and blue signal channels, respectively. (**b**) Spectral sensitivity of the *F* signal channel that is the product of the spectral sensitivity of a typical silicon sensor and the spectral transmittance of the Baader UV/IR cut filter.

The CMOS sensor converts the light into electric signals. Conventional CFA on the CMOS sensor filters light in order to separate the short-, mid- and long-wavelength components of the light. As shown in Figure 1a, the sensor pixels corresponding to the red, green and blue filters provide *R*, *G* and *B* signals, respectively. The spectral sensitivity vectors of the red, green and blue channels are designated with ***S***_CamR_, ***S***_CamG_ and ***S***_CamB_, respectively. Figure 2a shows the ***S***_CamR_, ***S***_CamG_ and ***S***_CamB_ of the D5100 camera measured using a monochromator [30]. The IR cutoff filter for the D5100 camera has a cutoff wavelength of approximately 690 nm. In this paper, it was assumed that the spectral sensitivities of the red, green and blue channels of the quadcolor cameras under consideration are the same as shown in Figure 2a.

Figure 2b shows the spectral sensitivity of the fourth channel of a quadcolor camera, which is the product of the spectral sensitivity of a typical silicon sensor [39] and the spectral transmittance of the Baader UV/IR cut filter. This fourth channel is the greenish yellow channel, although only the UV/IR cut filter is applied. The output signal of the channel is designated as the *F* signal because this channel is free of the optical filter. In order to distinguish it from the CIE stimulus Y, it is not designated as the *Y* signal. Therefore, the quadcolor camera with this fourth channel is called the RGBF camera.

**Figure 3:**
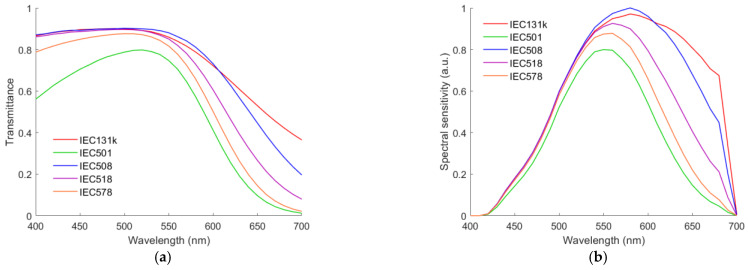
(**a**) Spectral transmittance of five compensation filters for camera sensors. (**b**) Spectral sensitivities of the yellowish green channels using the filters in (**a**).

Blue or cyan filters can be used to compensate for the increased sensitivity of silicon sensors with wavelength, e.g., Isuzu IEC series filters. Figure 3a shows the spectral transmittance of five IEC series filters. The spectral sensitivities of the channels with each of the five filters applied are shown in Figure 3b. They are the yellowish green channel. Because of the compensation filter applied, the quadcolor camera with such a fourth channel is called the RGBC camera.

**Figure 4:**
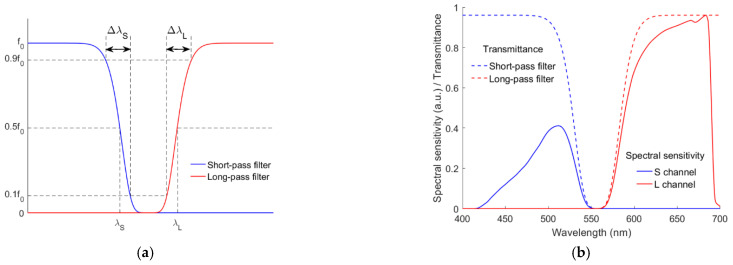
(**a**) Schematic diagram showing the specification definitions of the short-pass and long-pass optical filters. (**b**) Spectral sensitivity examples of the fourth channel using the short-pass and long-pass optical filters, where *λ*_S_ = 528 nm and *λ*_L_ = 585 nm. Refer to Section 2.1 for details.

Short-pass and long-pass optical filters were also applied to the fourth channel, respectively. The spectral transmittance of the optical filters is based on the super-Gaussian function. The spectral transmittance functions for the short-pass and long-pass optical filters are the same as for the cyan and yellow filters in [29], respectively. Figure 4a shows their specification, where *f*_0_ is the maximum transmittance; *λ*_S_ and *λ*_L_ are the edge wavelengths at 0.5 *f*_0_; Δ*λ*_S_ and Δ*λ*_L_ are the edge widths from 0.1 *f*_0_ to 0.9*f*_0_. In this paper, for simplicity, Δ*λ*_S_ = Δ*λ*_L_ = 30 nm were assumed. The fourth channel using the short-pass and long-pass optical filters are called the S and L channels, respectively. The quadcolor cameras with the S and L channels are called the RGBS and RGBL cameras, respectively. Figure 4b shows the spectral transmittance of the short-pass and long-pass optical filters with *λ*_S_ = 528 nm and *λ*_L_ = 585 nm, respectively, where the corresponding spectral sensitivities of the S and L channels are also shown.

To sum up, four types of quadcolor cameras were considered, which are the RGBF, RGBC, RGBS and RGBL cameras. They are identical except for the color filter applied to the fourth channel.

### 2.2. Color Samples

The reference/training and test samples were prepared using the reflectance spectra of matt Munsell color chips measured by a spectroradiometer [40]. A total of 1268 reflectance spectra in [40] were used in this paper. Illuminant D65 was assumed to be the light source. In the case of using the LUT method, the same 202 and 1066 color chips as in [29] were used to prepare the reference and test samples, respectively. In the case of using the wPCA method, the reference and test samples were also used as training and test samples, respectively.

The spectrum vector of the light reflected from a color chip is

(2) $$S_{\mathrm{Reflection}}=S_{\mathrm{Ref}}\circ S_{\mathrm{D65}},$$

where ***S***_Ref_ and ***S***_D65_ are the spectral reflectance vector of the color chip and the spectrum vector of the illuminant D65, respectively. The color points of light reflected from the 1268 Munsell color chips in the CIELAB color space have been shown in [29]. The CIE 1931 color-matching functions (CMFs) were adopted in this paper.

In the following, the RGBF camera is taken as an example. The measured signal of a color channel is *U*_Meas_ = ***S***_Reflection_^T^***S***_Cam*U*_, where *U* = *R*, *G*, *B* and *F* for the red, green, blue and fourth channels, respectively; ***S***_Reflection_ is the reflection spectrum vector; and ***S***_Cam*F*_ is the spectral sensitivity vector of the channel calculated from Equation (1). For the white balance condition, the channel signals are normalized to *U* = *U*_Meas_/*U*_MeasD65_, where *U* = *R*, *G*, *B* and *F*; *U*_MeasD65_ is the measured signal when ***S***_Ref_ = ***S***_White_, where ***S***_White_ is the spectral reflectance of a white card. The same white card in [29] was taken, which is the white side of a Kodak gray card.

**Figure 5:**
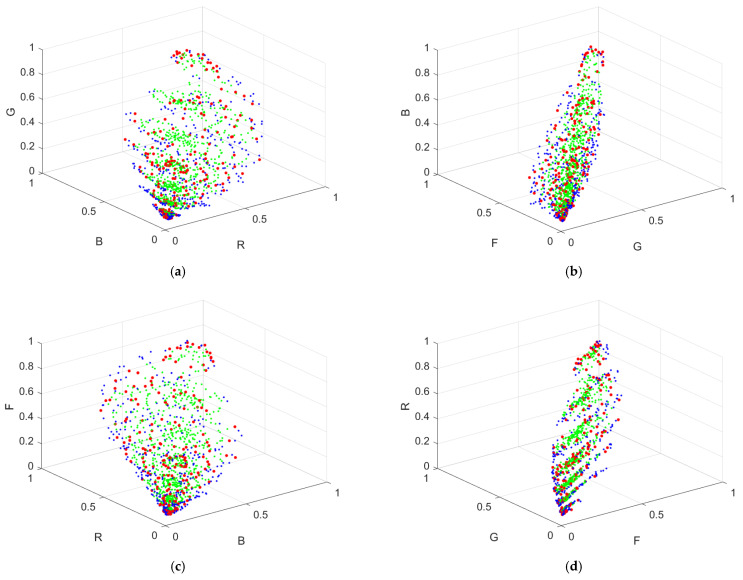
Color points of light reflected from the Munsell color chips in the (**a**) RGB, (**b**) GBF, (**c**) BFR and (**d**) FRG signal spaces, using the RGBF camera. The 202 reference samples are shown as red dots. The 726 inside samples and 340 outside samples are shown as green and blue dots, respectively.

The vector representing the camera signals is designated as ***C*** = [*R*, *G*, *B*, *F*]^T^. Figure 5a–d show the color points of the light reflected from the Munsell color chips in the RGB, GBF, BFR and FRG signal spaces, respectively, using the RGBF camera. In these figures, the 202 reference samples are shown as red dots; out of the 1066 test samples, the 726 inside samples and 340 outside samples are shown as green and blue dots, respectively.

### 2.3. Assessment Metrics

For a given test signal vector, the wPCA and LUT methods to reconstruct the reflection light spectrum are shown in Section 3.1 and Section 3.2, respectively. The reconstructed spectrum vector is designated as ***S***_Rec_. The reconstructed spectral reflectance vector ***S***_RefRec_ was calculated as the reflection spectrum vector ***S***_Rec_ divided by the D65 spectrum vector ***S***_D65_ element by element.

The reconstructed spectral reflectance vector ***S***_RefRec_ was assessed by the root mean square (RMS) error *E*_Ref_ *=* (|***S***_RefRec_ − ***S***_Ref_|^2^/*M*_w_)^1/2^ and the goodness-of-fit coefficient GFC = |***S***_RefRec_^T^***S***_Ref_|/|***S***_RefRec_||***S***_Ref_|, where |·| stands for the norm operation. The color difference between ***S***_Rec_ and ***S***_Reflection_ was assessed using CIEDE2000 Δ*E*_00_. The spectral comparison index (SCI) was also used to assess the reconstructed results, which is an index of metamerism [41]. The parameter *k* in the formula for calculating SCI shown in [41] was set to 1.0.

For the values of *E*_Ref_, Δ*E*_00_ and SCI, the smaller, the better. The statistics of the three metrics were calculated, which are the mean *μ*, standard deviation *σ*, 50th percentile PC50, 98th percentile PC98 and maximum MAX. For the value of GFC, the larger, the better. The statistics of GFC were calculated, which are the mean *μ*, standard deviation *σ*, 50th percentile PC50, and minimum MIN. The fit of the spectral curve shape is good if GFC > 0.99 [28,42]. The ratio of samples with GFC > 0.99 was calculated, which is called the ratio of good fit and designated as RGF99.

The assessment metrics *E*_Ref_ and 1– GFC are related to spectral error. The assessment metrics Δ*E*_00_ and SCI are related to color appearance error. Section 4.3 will show that the values of *E*_Ref_ and 1 − GFC are roughly consistent with each other; the values of Δ*E*_00_ and SCI are also roughly consistent with each other. Since the reconstructed spectrum is a metameric spectrum, the spectral error can be large when the color appearance error is small. Therefore, it is necessary to use these two types of metrics for assessment.

## 3. Methods

### 3.1. The wPCA Method

From the theory of PCA [43], the spectrum vector ***S*** can be decomposed as

(3) $$S=P_{0}+\sum_{k=1}^{M_{w}}d_{k}k_{\;},$$

where ***P***_0_ is the average spectrum vector of training samples; *d_k_* and ***P****_k_* are the coefficient and spectrum vector of the *k*-th principal component. Principal components are derived from training samples using PCA. The number of principal components is the number of sampling wavelengths, *M_w_*. The camera spectral sensitivity matrix is defined as ***D***_Cam_ = (***D***_CamR_ ***D***_CamG_ ***D***_CamB_ ***D***_Cam4th_), where ***D***_CamR_, ***D***_CamG_, ***D***_CamB_ and ***D***_Cam4th_ are the normalized spectral sensitivity vectors of the red, green, blue and fourth channel for the white balance condition. For example, ***D***_CamR_ = ***S***_CamR_/(***S***_White_∘***S***_D65_)^T^ ***S***_CamR_. ***D***_Cam_ is an *M*_w_ × 4 matrix. If both sides of Equation (3) are multiplied by ***D***_Cam_^T^, we have the signal vector

(4) $$C=C_{0}+\sum_{k=1}^{M_{w}}d_{k}k_{\;},$$

where ***C***_0_ = ***D***_Cam_^T^***P***_0_ and ***Q****_k_* = ***D***_Cam_^T^***P****_k_*. Since Equation (4) represents four scalar equations, the summation in Equation (4) was truncated, and the upper limit of the summation index *M*_w_ was modified to 4 for solving the first four *d_k_* coefficients. From the solved coefficients, the reconstructed spectrum vector is

(5) $$S_{\mathrm{Rec}}=P_{0}+\sum_{k=1}^{4}d_{k}k_{\;}.$$

If the reconstructed spectrum has negative values, the value is set to zero. The first four principal components are the basis spectra for the spectrum reconstruction using a quadcolor camera. The channel spectral sensitivity vectors are given in Section 2.1. As described in Section 1, in practice, the spectral sensitivity matrix ***D***_Cam_ is measured or estimated experimentally. Additional errors introduced from the measurements/estimations were not considered in this paper.

The wPCA method is the same as the PCA method shown above, except that training samples are weighted according to the sample to be reconstructed [22]. The *i*-th training sample was multiplied by a weighting factor Δ*E_i_* ^−^ ^*γ*^, where Δ*E_i_* is the color difference between the test sample and the *i*-th training sample in CIELAB color space; *γ* is a constant. Weighted training samples were used to derive basis spectra. The larger the value of *γ*, the smaller the color difference, and the greater the contribution of the training samples to the basis spectra. If *γ* = 0, the wPCA method becomes the traditional PCA method. The value of *γ* is usually set to 1.0 [22]. The value of *γ* was optimized for the minimum mean *E*_Ref_ of the test samples for individual camera in this paper. A camera device model was used to convert RGB signal values into tristimulus values for calculating Δ*E_i_*. A third-order root polynomial regression model (RPRM) was employed and trained using the reference samples [44]. The accuracy of the RPRM was slightly higher than that of the polynomial regression model in this case.

We also tried to use the weighting factor GFC*_i_* *^κ^* instead of Δ*E_i_^−γ^*, where *κ* is a constant to be optimized; GFC*_i_* is the GFC of the test sample and the *i*-th training sample. The larger the value of *κ*, the larger the goodness-of-fitting coefficient, and the greater the contribution of the training samples to the basis spectra. Using such a weighting factor requires two-stage spectrum reconstruction. The first stage reconstructs the spectrum using the weighting factor Δ*E_i_^−γ^*. The reconstruct spectrum is used to calculate the GFC*_i_*. The second stage reconstructs the spectrum using the weighting factor GFC*_i_^κ^*. However, the spectrum reconstruction error using the weighting factor GFC*_i_^κ^* is larger than using the weighting factor Δ*E_i_^−γ^*. Therefore, this paper only considers the case of using the weighting factor Δ*E_i_^−γ^*.

### 3.2. The LUT Method: Interpolation

Detailed descriptions of the LUT method for the 3D case were given in [25,29]. The LUT method for the 4D case is the same as for the 3D case, but the signal dimensions are different. This subsection shows the reconstruction of reflection spectrum vector ***S***_Rec_ from the test signal vector ***C*** for the 4D case. Linear scattered data interpolation was used to reconstruct the spectrum due to its simplicity and computational time savings [25,26,27,29]. A simplex mesh in the signal space was generated from the reference signal vectors using the Delaunay triangulation [25]. For example, a simplex is the triangle and tetrahedron in 2D and 3D signal spaces, respectively. All programs for this work were implemented in MATLAB (version R2021a, MathWorks). The simplex mesh was generated by the MATLAB function “delaunayn” [45]. There were three steps to interpolate the test sample.

(i) The simplex that encloses the vector ***C*** in the signal space was located. This paper used the MATLAB function “tsearchn” to locate the simplex [46].
(ii) It is required that ***C*** is the linear combination of the five reference signal vectors at the vertices of the simplex, and
  ***C*** = *α*_1_***C***_1_ + *α*_2_***C***_2_ + *α*_3_***C***_3_ + *α*_4_***C***_4_ + *α*_5_***C***_5_, (6)
  1 = *α*_1_ + *α*_2_ + *α*_3_ + *α*_4_ + *α*_5_, (7)
  where the coefficients *α*_1_, *α*_2_, *α*_3_, *α*_4_ and *α*_5_ are weighting factors. Equation (6) comprises four scalar equations because the signal vector is 4D. Equation (7) guarantees that the color point of the signal vector is inside the simplex in the signal space if 0 < *α*_1_, *α*_2_, *α*_3_, *α*_4_, *α*_5_ < 1. The five coefficients in Equations (6) and (7) were solved.
(iii) The reconstructed reflection spectrum vector is
  ***S***_Rec_ = *α*_1_***S***_1_ + *α*_2_***S***_2_ + *α*_3_***S***_3_ + *α*_4_***S***_4_ + *α*_5_***S***_5_, (8)
  where ***S****_j_* is the reference spectrum vector corresponding to the *j*-th vertex, *j* = 1, 2, 3, 4 and 5. If the reconstructed spectrum has negative values, the value is set to zero.

The solutions to the coefficients in Equations (6) and (7) are unique. These coefficients are called barycentric coordinates. They describe the location of the color point in the simplex [25]. The linear interpolation is called the barycentric interpolation.

If a signal vector is outside the convex hull of the simplex mesh, it is an outside sample. Figure 5a–d show the 340 outside samples as blue dots in the signal space using the RGBF camera. The method to extrapolate outside samples is described in Section 3.3.

### 3.3. The LUT Method: Extrapolation

Section 2.2 shows that there are 340 outside samples using the RGBF camera, while the number of outside samples is 202 using the D5100 camera [29] for the same 202 reference samples and 1066 test samples. Imagine projecting multiple points in a 3D space onto a 2D space. If the point volume density in the 3D space is low, the point density in the 2D space can be high and vice versa. Similarly, if the color point density of the reference samples in the RGB signal space is high enough to interpolate, say, 80% of the test samples, then the color point density of the same reference samples in the 4D signal space may only be able to interpolate, say, 70% of the test samples. Therefore, for the same reference and test samples, the number of outside samples increases with the signal dimension.

The spectra of all signals can be reconstructed using the wPCA method but not the LUT method. However, due to the lack of suitable nearby training samples, as shown in Figure 5a–d, the spectrum reconstruction error of an outside sample using the wPCA method is likely to be larger than that of an inside sample. In this paper, outside samples of the LUT method were extrapolated utilizing the reference samples and ARSs [29]. ARSs are high-saturation samples. They are created using appropriately selected color filters and color chips. The color filters are called the ARS filters. The extrapolation process is the same as the interpolation method shown in Section 3.2 but using the expanded reference samples including ARSs.

The authors of [29] used cyan, magenta and yellow (CMY) ARS filters to extrapolate the 202 outside samples for the case with the D5100 camera. It was found that of the 340 outside samples, a few cannot be extrapolated using the CMY ARS filters for some quadcolor cameras under consideration. For example, 2 outside samples cannot be extrapolated using the RGBC cameras. It was also found that the use of additional red, green, and blue (RGB) ARS filters to create more ARSs enables all outside samples to be extrapolated.

**Figure 6:**
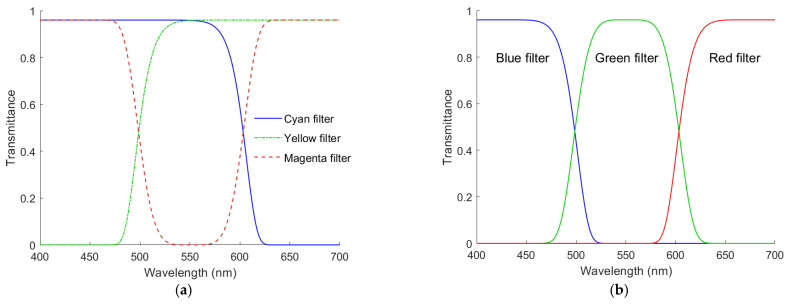
Spectral transmittance of the (**a**) cyan, yellow, magenta, (**b**) red, green and blue filters for creating ARSs. Refer to Section 3.3 for details.

The ARS filters can be optimized to minimize spectrum reconstruction errors, but the optimization requires the spectral sensitivity functions of the camera. Filter characteristics can be specified by the edge wavelength and edge width, which are defined in the same way as the color filter of the fourth channel in Section 2.1. Although the design is not optimal, their specifications can be selected according to channel wavelengths. The channel wavelength is the average wavelength of the spectral sensitivity of the signal channel. The RGB channel wavelengths of the D5100 camera are λ_CamR_ = 603.4 nm, λ_CamG_ = 530.7 nm and λ_CamB_ = 466.7 nm, respectively. From [29], empirically, the edge wavelengths of cyan and yellow filters can be λ_C_ = λ_CamR_ and λ_Y_ = λ_CamC_, respectively, where λ_CamC_ = (λ_CamB_ + λ_CamG_)/2 is the mean wavelength of λ_CamB_ and λ_CamG_; the edge wavelengths at the short-wavelength side and the long-wavelength side of the magenta filter can be *λ_MS_* = λ_CamC_ and *λ_ML_* = λ_CamR_, respectively. Therefore, we set λ_C_ = 603.4 nm, λ_Y_ = 498.7 nm, *λ_MS_* = 498.7 nm and *λ_ML_* = 603.4 nm. Figure 6a shows the spectral transmittance of the CMY ARS filters, where the maximum transmittance and edge width of all filters were set to 0.9 and 30 nm, respectively.

The spectral transmittance of the RGB ARS filters is also based on the super-Gaussian function. In [29], the spectral transmittance function of the magenta ARS filter is an inverted super-Gaussian function. Since filter optimization is not the purpose of this paper, for simplicity, we set the edge wavelengths of the blue and red filters as λ_B_ = λ_CamC_ and λ_R_ = λ_CamR_, respectively; the edge wavelengths on the short- and long-wavelength sides of the green filter were *λ_GS_* = λ_CamC_ and *λ_GL_* = λ_CamR_, respectively. Therefore, we set λ_R_ = 603.4 nm, λ_B_ = 498.7 nm, *λ_GS_* = 498.7 nm and *λ_GL_* = 603.4 nm. Figure 6b shows the spectral transmittance of the RGB ARS filters, where the maximum transmittance and edge width of all filters were set to 0.9 and 30 nm, respectively. The CMY and RGB filters are designated as the CMYRGB filters.

**Figure 7:**
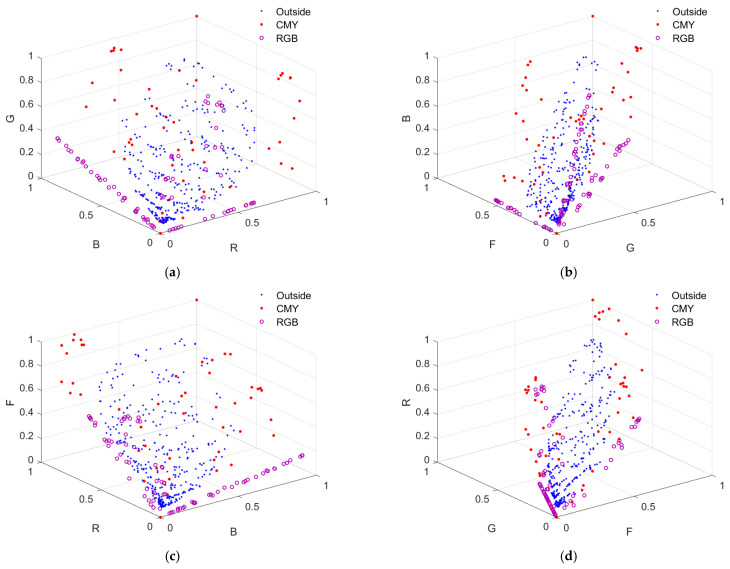
Color points of ARSs created with CYM and RGB filters shown as 47 red dots and 79 purple hollow dots, respectively, in the (**a**) RGB, (**b**) GBF, (**c**) BFR and (**d**) FRG signal spaces using the RGBF camera. The 340 outside samples are shown as blue dots for comparison.

The ARSs were created according to the method in [29] using the CMYRGB filters specified above and the color chips corresponding to the vertices of the reference sample convex hull. The convex hull in the RGBF signal space cannot be shown due to its 4D geometry. Figure 7a–d show the color points of the ARSs created with the CYMRGB filters in the RGB, GBF, BFR and FRG signal spaces, respectively, for the case with the RGBF camera. The color points of ARSs created using CYM and RGB filters are shown as 47 red dots and 79 purple hollow dots, respectively. Figure 7a–d also show the 340 outside samples as blue dots for comparison. We can see that the gamut volume expanded by the ARSs in Figure 7a–d is larger than that expanded by the reference samples in Figure 5a–d. Both the reference samples and the ARSs created with the CMYRGB filters were used to extrapolate the outside samples for the cases with the tricolor and quadcolor cameras under consideration using the LUT method. The inclusion of the ARSs in the training sample set was found to deteriorate the spectrum reconstruction using the wPCA method as in [29].

### 3.4. CMF Mismatch Factor (CMFMisF)

If both sides of Equation (8) are multiplied by the spectral sensitivity matrix ***D***_Cam_^T^ and integrated over the wavelength, we obtain Equation (6). However, the interpolation is an inverse problem. The reconstructed spectrum vector ***S***_Rec_ is one of numerous metameric spectrum vectors corresponding to the test signal vector ***C***. Equations (6) and (7) are five constraints for finding a metameric spectrum vector. If a tricolor camera is used to reconstruct the spectrum, the number of constraints is only four. The difference between the target spectral reflectance vector ***S***_Ref_ and the reconstructed spectral reflectance vector ***S***_RefRec_ calculated from the metameric spectrum vector ***S***_Rec_ was assessed using the metrics defined in Section 2.3.

Appendix A and Appendix B show zero-color-difference conditions using the LUT and wPCA methods, respectively. If the sensitivity functions of the camera fit the CIE CMFs, $\bar{x}$, $\bar{y}$ and $\bar{z}$, ideally, the color difference between the reflection light spectrum ***S***_Reflection_ and the spectrum ***S***_Rec_ reconstructed using the LUT method is zero. Under this condition, the tristimulus XYZ values of the metameric spectrum vector ***S***_Rec_ are the same as those of the sample to be reconstructed. For the case of using the wPCA method, the color difference is also zero if the additional condition is satisfied, which requires that the spectral amplitude calculated from Equation (5) be non-negative.

The color difference Δ*E*_00_ between ***S***_Reflection_ and ***S***_Rec_ will be non-zero when the spectral sensitivity functions of the considered cameras does not fit the CMFs ideally. Therefore, it can be inferred that the mean Δ*E*_00_ of the test samples is related to the CMF mismatch factor (CMFMisF) defined as

CMFMisF *=* (*σ_x_*^2^ + *σ_y_*^2^ + *σ_z_*^2^)^1/2^, (9)

where *σ_m_* is the relative RMS error of fitting the CMF vector ***u***_m_ using the spectral sensitivity vectors for *m* = *x*, *y*, and *z*; ***u****_x_* = [$\bar{x}$(*λ*_1_),$\bar{x}$(*λ*_2_), …,$\bar{x}$(*λ_M_*_w_)]^T^, ***u****_y_* = [$\bar{y}$(*λ*_1_),$\bar{y}$(*λ*_2_), …, $\bar{y}$(*λ_M_*_w_)]^T^, ***u****_z_* = [$\bar{z}$(*λ*_1_), $\bar{z}$(*λ*_2_), …, $\bar{z}$(*λ_M_*_w_)]^T^ and *λ_j_* = *λ*_1_ + (*j* –1) Δ*λ* for *j* = 1, 2, …, *M*_w_.

*σ_m_* = (|***u***_Fit_ − ***u****_m_*|^2^ )^1/2^/|***u****_m_*|, (10)

where *m* = *x*, *y*, and *z*; ***u***_Fit_ is the least squares fit of the CMF vector ***u****_m_* using the spectral sensitivity vectors of the camera. For the case of using the quadcolor camera,

***u***_Fit_ = *β*_R_***S***_CamR_ + *β*_G_***S***_CamG_ + *β*_B_***S***_CamB_ + *β*_4th_***S***_Cam4th_, (11)

where the coefficients *β*_R_, *β*_G_, *β*_B_ and *β*_4th_ were solved using the Moore–Penrose pseudo-inversion in least-squares sense [43].

## 4. Results and Discussion

### 4.1. Using the RGBF Camera

Table 1 shows the assessment metric statistics for the test samples using the LUT method, where the cameras are the D5100 and RGBF. The LUT method for the D5100 camera is the same as the quadcolor camera shown in Section 3.2, except that the signal space is reduced from 4D to 3D. As can be seen from Table 1, using the RGBF camera reduced the mean *E*_Ref_, Δ*E*_00_ and SCI of the inside samples and increased the mean GFC of the inside samples compared to the D5100 camera. The mean *E*_Ref_ and GFC values of the outside samples using the RGBF camera were even smaller and larger than the inside samples using the D5100 camera, respectively. While non-zero as expected, the color difference Δ*E*_00_ was small for most of the test samples. Compared to the D5100 camera, the mean *E*_Ref_ of the test samples, inside samples and outside samples using the RGBF camera was reduced by 31.98%, 35.81% and 35.82%, respectively. Compared to the D5100 camera, the RGF99 of the test samples, inside samples and outside samples using the RGBF camera was increased from 0.9343, 0.9375 and 0.9208 to 0.9887, 0.9972 and 0.9706, respectively.

Table 2 is the same as Table 1 except that the wPCA method was used. The wPCA method for the D5100 camera is the same as the quadcolor camera shown in Section 3.1, except that three basis spectra were used. The inside and outside samples using the wPCA method were the same as those using the LUT method for comparison. The optimized *γ* = 1.7 and 1.2 for the cases of using the D5100 and RGBF cameras, respectively. From Table 2, the mean assessment metrics of the outside samples were worse than those of the inside samples. Compared to the D5100 camera, the mean *E*_Ref_ values of the test samples, inside samples and outside samples using the RGBF camera were reduced by 21.6%, 27.3% and 24.9%, respectively. Compared to the D5100 camera, the RGF99 values of the test samples, inside samples and outside samples using the RGBF camera increased from 0.9493, 0.9676 and 0.8713 to 0.9765, 0.9945 and 0.9382, respectively. The improvement of RGF99 on outside samples using the RGBF camera is significant.

**Figure 8:**
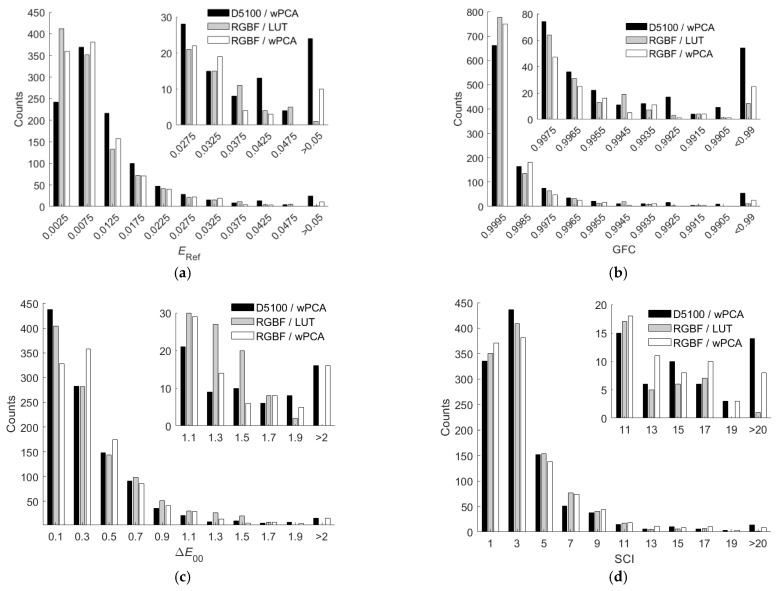
(**a**) *E*_Ref_, (**b**) GFC, (**c**) Δ*E*_00_ and (**d**) SCI histograms for the 1066 test samples. The corresponding camera and spectrum reconstruction method are shown in figures. The insets in (**a**–**d**) show enlarged parts.

From Table 1 and Table 2, it can be seen that the LUT method outperformed the wPCA method using the RGBF camera. Note that the wPCA method outperformed the LUT method using the D5100 camera except for about two orders of magnitude longer computation time [27,29]. In [29], the LUT method outperformed the wPCA method using the D5100 camera because the value of *γ* was not optimized for the wPCA method, where *γ* = 1.0. Figure 8a–d show the *E*_Ref_, GFC, Δ*E*_00_, and SCI histograms for the test samples, respectively, where the three shown cases are (i) using the D5100 camera and the wPCA method, (ii) using the RGBF camera and the LUT method, and (iii) using the RGBF camera and the wPCA method.

From Figure 8a–d, the numbers of test samples in the “*E*_Ref_ > 0.05”, “GFC < 0.99”, “Δ*E*_00_ > 2.0”, and “SCI > 20” bins using the LUT method are less than those using the wPCA method. From Table 1 and Table 2, using the RGBF camera, the maximum *E*_Ref_ = 0.0567 and 0.0742 for the cases of using the LUT and wPCA method, respectively. These results show that using the LUT method is more reliable for spectral reflectance recovery. However, when using the LUT method or the wPCA method, the assessment metrics were improved using the RGBF camera compared to the D5100 camera.

**Figure 9:**
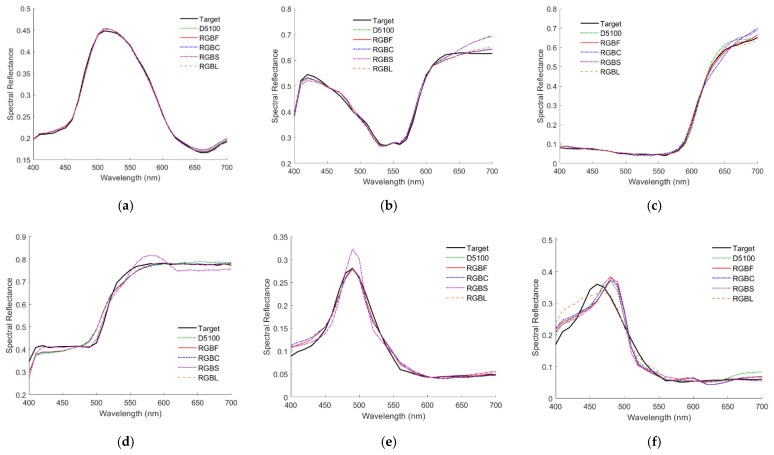
The target spectra ***S***_Ref_ and recovered reflectance spectra ***S***_RefRec_ of the test samples with the (**a**) 2.5G 7/6, (**b**) 10P 7/8, (**c**) 2.5R 4/12, (**d**) 2.5Y 9/4, (**e**) 10BG 4/8 and (**f**) 5PB 4/12 color chips. The cameras are indicated. Spectra are reconstructed using the LUT method.

**Figure 10:**
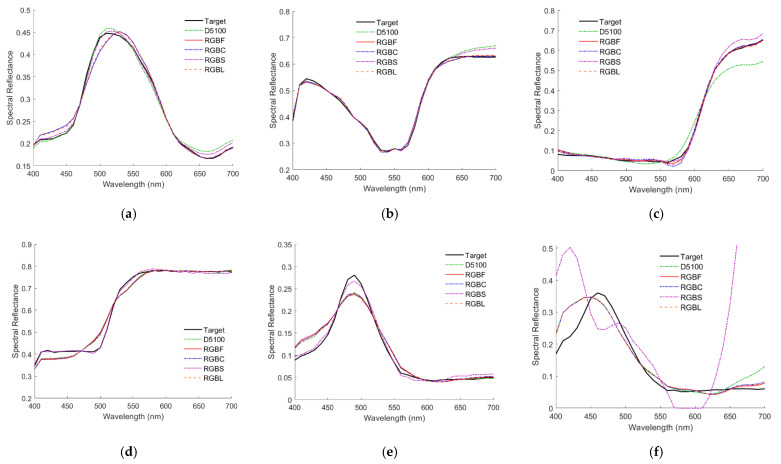
(**a**–**f**) are the same as Figure 9a–f respectively, except that spectra were reconstructed using the wPCA method.

Since the mean assessment metrics of outside samples are worse than those of inside samples, the spectrum reconstruction of outside samples was investigated in more detail. Figure 9a–f show the recovered spectral reflectance ***S***_RefRec_ using the LUT method from the light reflected from 2.5G 7/6, 10P 7/8, 2.5R 4/12, 2.5Y 9/4, 10BG 4/8 and 5PB 4/12 color chips, respectively, where their target reflectance ***S***_Ref_ values are also shown. In addition to the D5100 and RGBF cameras, Figure 9a–f also show the results using other cameras, which will be considered in the following subsections. The same color chips were used as examples in [29] to show the spectral reflectance recovery using the D5100 camera and the LUT method. All the cases in Figure 9a–f are outside examples. The case in Figure 9a is an inside sample using the D5100 camera, but it becomes an outside sample using the RGBF camera. Figure 10a–f are the same as Figure 9a–f, respectively, except that spectra were recovered using the wPCA method.

Table 3 shows the *E*_Ref_ values for the cases shown in Figure 9a–f and Figure 10a–f. Table 4 is the same as Table 3 except that the values of Δ*E*_00_ are shown. Values for *E*_Ref_ and Δ*E*_00_ larger than 0.03 and 1.0, respectively, are shown in bold. The cases where the error *E*_Ref_ of using the RGBF camera is larger than that of using the D5100 camera are the cases of Figure 9e,f and the case of Figure 10a. The cases where the difference Δ*E*_00_ of using the RGBF camera is larger than that of using the D5100 camera are the cases of Figure 9b,f and the cases of Figure 10a,b,f. Compared to the D5100 camera, using the RGBF camera effectively improved the statistics of the assessment metrics, but it does not guarantee better spectral reflectance recovery for every color chip tested.

### 4.2. Using the RGBC Camera

Since there are red, green and blue channels, the fourth channel is reasonably designed to be either a cyan channel or a yellow channel. From the spectral sensitivity of the silicon sensor shown in Figure 2b, if the fourth channel is a cyan channel, its sensitivity in long wavelength is suppressed. Using a compensation color filter shown in Figure 3a modifies the greenish yellow channel in Figure 2b to a yellowish green channel in Figure 3b instead of a cyan channel. If the applied color filter has a much smaller mid- and long-wavelength transmittance than the filters shown in Figure 3a, the fourth channel becomes the blue or greenish blue channel. The S channel of the RGBS camera is an example of such a case, which will be considered in Section 4.3. The case with spectral sensitivity shown in Figure 2b has been considered in Section 4.1, i.e., the RGBF camera. This subsection will consider the cases with spectral sensitivities shown in Figure 3b.

**Figure 11:**
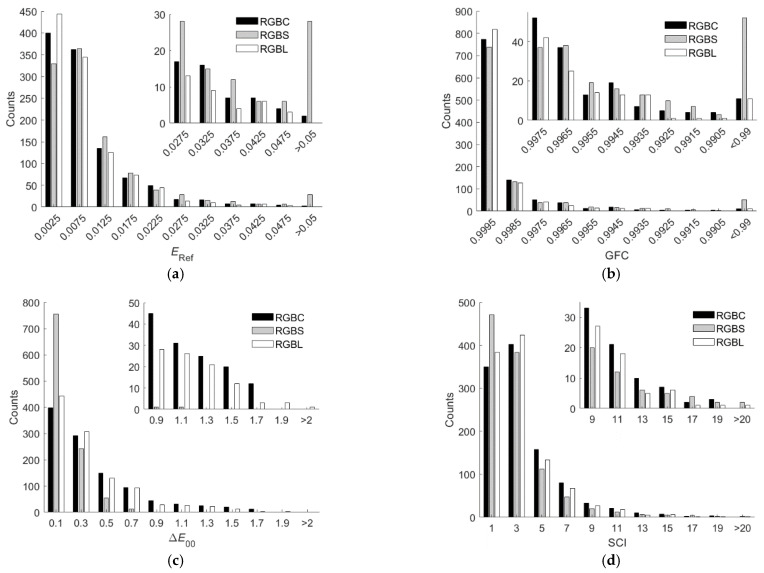
(**a**) *E*_Ref_, (**b**) GFC, (**c**) Δ*E*_00_ and (**d**) SCI histograms for the 1066 test samples. The cameras are the optimized RGBC, RGBS and RGBL cameras. Spectra are reconstructed using the LUT method. The insets in (**a**–**d**) show enlarged parts.

**Figure 12:**
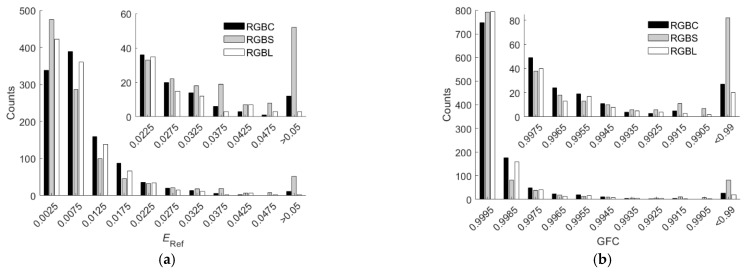

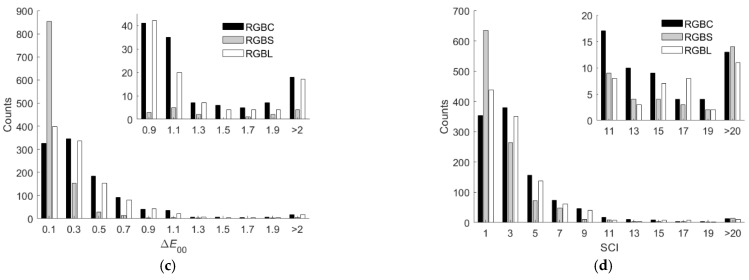
The same as Figure 11 except that spectra were reconstructed using the wPCA method. (**a**) *E*_Ref_, (**b**) GFC, (**c**) Δ*E*_00_ and (**d**) SCI histograms for the 1066 test samples.

It was found that among the five color filters in Figure 3a, the spectrum reconstruction error was the smallest when the IEC 131K filter was applied to the fourth channel. Using the LUT method, the mean *E*_Ref_ = 0.0091, 0.0128, 0.0093, 0.0099 and 0.0125 for the cases with the IEC 131K, 501, 508, 518 and 578 filters, respectively; RGF99 = 0.9897, 0.9060, 0.9878, 0.9803 and 0.9240 for the cases with the IEC 131K, 501, 508, 518 and 578 filters, respectively. The mean spectrum reconstruction error increased as the spectral transmittance of filter decreased in the long wavelength region. Table 5 shows the assessment metric statistics for the test samples of the RGBC camera with the IEC 131K filter using the LUT and wPCA methods. For the case of using the wPCA method, the optimized *γ* = 1.2. Figure 11a–d show the *E*_Ref_, GFC, Δ*E*_00_, and SCI histograms, respectively, for the test samples of the optimized RGBC camera with the IEC 131K filter, using the LUT method. Figure 12a–d are the same as Figure 11a–d, except for using the wPCA method. Figure 9a–f and Figure 10a–f also show the spectral reflectance recovery examples using the optimized RGBC camera, where the values of *E*_Ref_ and Δ*E*_00_ are shown in Table 3 and Table 4, respectively.

For ease of comparison, the assessment metric statistics for using the RGBF camera are also shown in Table 5. As can be seen from Table 5, the assessment metric statistics using the optimized RGBC camera were about the same as the RGBF camera but with an additional compensation color filter. The use of the compensation color filter to suppress the spectral sensitivity of the fourth channel in the long wavelength region did not improve the performance of spectrum reconstruction.

### 4.3. Using the RGBS and RGBL Cameras

The above results show that using the fourth channel without a compensation color filter produced a slightly smaller mean *E*_Ref_. Further reduction in the mean *E*_Ref_ is possible if the spectral sensitivity of the fourth channel can be appropriately modified using a color filter. This subsection considers the cases of using the short-pass and long-pass filters defined in Section 2.1 as the color filter applied to the fourth channel.

**Figure 13:**
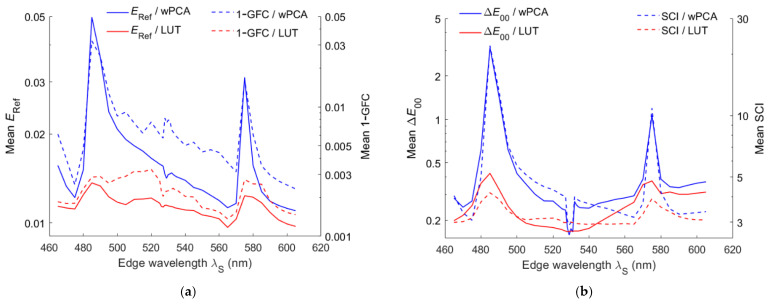
Mean (**a**) *E*_Ref_, 1 – GFC and (**b**) Δ*E*_00_, SCI versus the edge wavelength of the short-pass optical filter using the wPCA and LUT methods.

**Figure 14:**
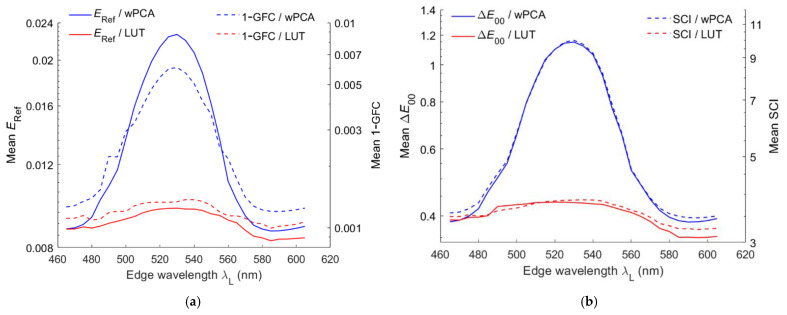
The same as Figure 13 except a long-pass optical filter is used. Mean (**a**) *E*_Ref_, 1 – GFC and (**b**) Δ*E*_00_.

**Figure 15:**
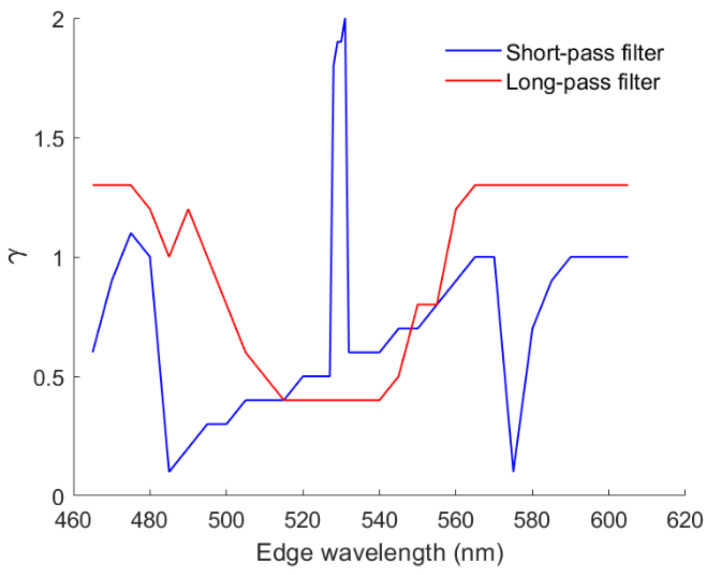
The optimized values of *γ* for the case of using the wPCA method in Figure 13 and Figure 14.

Figure 13a shows the mean *E*_Ref_ and 1– GFC of the test samples using the LUT and wPCA methods versus the edge wavelength *λ*_S_ of the short-pass optical filter. Figure 13b is the same as Figure 13a except that the mean Δ*E*_00_ and SCI are shown. Figure 14a,b are the same as Figure 13a,b, respectively, except that the long-pass optical filter was used. As can be seen from Figure 13a and Figure 14a, the mean 1– GFC roughly followed the mean *E*_Ref_. From Figure 13b and Figure 14b, the mean SCI roughly followed the mean Δ*E*_00_. Figure 15 shows the optimized value of *γ* for the case of using the wPCA method in Figure 13 and Figure 14. For the case of using the RGBS camera, the optimized value of *γ* for the wPCA method is larger around 530 nm, as shown in Figure 15. We will show that the minimum CMFMisF is at this wavelength. From Figure 13b and Figure 14a, it can be seen that the RGBS and RGBL cameras are suitable designed for low color difference and low spectral error, respectively.

From Figure 13a,b, using the LUT method, the quadcolor camera optimized for the minimum mean Δ*E*_00_ is the RGBS camera using the short-pass optical filter of *λ*_S_ = 528 nm. For this optimized RGBS camera, the mean *E*_Ref_ = 0.0115 and Δ*E*_00_ = 0.1666, where the mean *E*_Ref_ is larger than the RGBF camera but smaller than the D5100 camera; the mean Δ*E*_00_ is much smaller than the RGBF and D5100 cameras; the maximum Δ*E*_00_ is only 1.0169. Using the wPCA method, the edge wavelength of the optimized RGBS is *λ*_S_ = 529 nm and the optimized *γ* = 1.9. For this optimized RGBS camera, the mean *E*_Ref_ = 0.0141 and Δ*E*_00_ = 0.158, where the mean *E*_Ref_ and Δ*E*_00_ are larger and smaller, respectively, than the D5100 and RGBF cameras.

From Figure 14a,b, using the LUT method, the quadcolor camera optimized for the minimum mean *E*_Ref_ is the RGBL camera using the long-pass optical filter of *λ*_L_ = 585 nm. The mean *E*_Ref_ = 0.0083 and Δ*E*_00_ = 0.3506 using the optimized RGBL camera with *λ*_L_ = 585 nm are smaller than the mean *E*_Ref_ = 0.0089 and Δ*E*_00_ = 0.3992 using the RGBF camera, respectively. Using the wPCA method, the edge wavelength of the optimized RGBL is *λ*_L_ = 587 nm and the optimized *γ* = 1.3. The mean *E*_Ref_ = 0.0087 and Δ*E*_00_ = 0.3862 using the optimized RGBL camera are smaller than the mean *E*_Ref_ = 0.0095 and Δ*E*_00_ = 0.4257 using the RGBF camera, respectively.

For the cases of using the LUT method, the spectral transmittance of the optimized long-pass and short-pass optical filters and their corresponding fourth channel spectral sensitivities are shown in Figure 3b. The histograms of assessment metrics for using the optimized RGBS and RGBL cameras are shown in Figure 11a–d for the case of using the LUT method. Figure 12a–d are the same as Figure 11a–d, respectively, except for the case of using the wPCA method. From Figure 11a–d and Figure 12a–d, it can be seen that the histogram characteristics for the RGBC and RGBL cameras are similar when using the LUT method or the wPCA method. The histogram characteristics for the RGBS camera are quite different from those for the RGBC and RGBL cameras when using the LUT method or the wPCA method. For the case of using the RGBS camera and the LUT method, there are 28, 52 and 2 test samples in the “*E*_Ref_ > 0.05”, “GFC < 0.99” and “SCI > 20” bins, respectively, while there is only 1 test sample in the “Δ*E*_00_ = 1.1” bin and no test sample in the larger Δ*E*_00_ bins. For this case, the color difference of all test samples is low despite the large spectral error. For the case of using the RGBS camera and the wPCA method, there are 52, 82, 4 and 14 test samples in the “*E*_Ref_ > 0.05”, “GFC < 0.99”, “Δ*E*_00_ > 2.0” and “SCI > 20” bins, respectively. For this case, most of the test samples have a low color difference, but there are few test samples with a large color difference. Therefore, if the RGBS camera is used as an imaging colorimeter, it is more reliable to reconstruct spectra using the LUT method.

Table 5 shows the assessment metric statistics for the test samples of the optimized RGBS and RGBL cameras using the LUT and wPCA methods. For the case of using the LUT or wPCA method, the mean *E*_Ref_ and mean Δ*E*_00_ using the optimized RGBS camera are the largest and smallest, respectively, compared to the other quadcolor cameras. Figure 9a–f and Figure 10a–f also show the spectral reflectance recovery examples using the optimized RGBS and RGBL cameras, where the values of *E*_Ref_ and Δ*E*_00_ are shown in Table 3 and Table 4, respectively. Notably, Figure 10f shows poor spectral reflectance recovered using the RGBS camera and the wPCA method, where the reflectance is 1.486 at 700 nm and zero around 580 nm. The zero is due to the negative value calculated from Equation (5).

**Figure 16:**
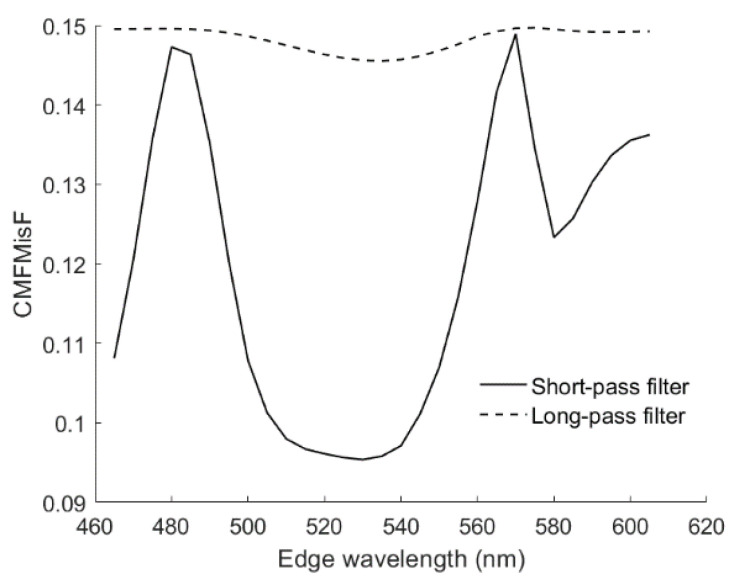
CMF mismatch factor CMFMisF versus the edge wavelength of the optical filter.

**Figure 17:**
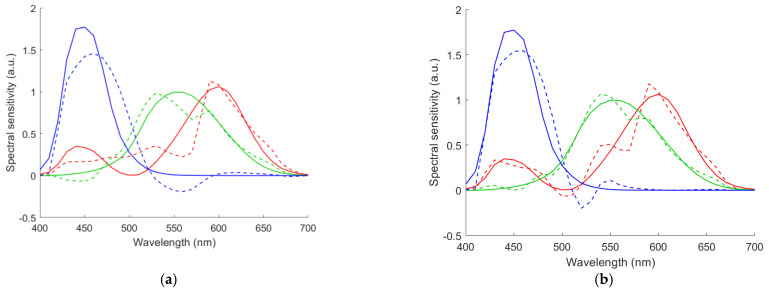
Least squares fit of CMF vectors using the spectral sensitivity vectors of (**a**) the RGBF camera and (**b**) the optimized RGBS camera with the 528 nm short-pass optical filter. The CMFs $\bar{x}$, $\bar{y}$ and $\bar{z}$ are shown in red, green and blue, respectively. Solid and dash lines show the CMFs and least squares fits, respectively.

The small color difference using the optimized RGBS camera can be explained using the CMFMisF defined in Section 3.4. Figure 16 shows the CMFMisF value versus the edge wavelength of the optical filter. Comparing Figure 16 with Figure 13b and Figure 14b, it can be seen that the mean Δ*E*_00_ closely relates to CMFMisF for the RGBS camera. The optimized edge wavelength of the RGBS camera using the LUT method or the wPCA method is about the wavelength of the minimum value in Figure 16, where the minimum CMFMisF = 0.09537 at *λ*_S_ = 530 nm. CMFMisF = 0.1495, 0.1495, 0.1486, 0.09543 and 0.1493 for the RGB, RGBF, optimized RGBC, optimized RGBS (*λ*_S_ = 528 nm) and optimized RGBL (*λ*_L_ = 585 nm) cameras, respectively. Note that the CMFMisF values are the same for the RGB and RGBF cameras, since the fourth channel of the RGBF camera contributes negligibly to the fit. The CMFs can be better fitted using the spectral sensitivities of the optimized RGBS camera. Figure 17a,b show the least squares fits of the CMF vectors using the spectral sensitivity vectors of the RGBF and optimized RGBS camera (*λ*_S_ = 528 nm), respectively. Although the CMF vectors were not well fitted in Figure 17a, if the spectrum of the test sample is well reconstructed using the RGBF camera, the color difference Δ*E*_00_ of the test sample will not be small.

As a comparison, the authors of [29] showed that for the same test samples using the CMF camera and optimized CMY ARS filters, the mean *E*_Ref_, GFC, Δ*E*_00_ and SCI are 0.0132, 0.9972, 0.0 and 4.1869, respectively. The CMF camera is the artificial tricolor camera with spectral sensitivity functions that are the same as the CMFs. While the mean Δ*E*_00_ is zero, the mean *E*_Ref_ of the CMF camera is about the same as the D5100 camera.

### 4.4. Cross Comparison

From the results shown above, the LUT method outperformed the wPCA method in spectrum reconstruction. We first discuss the case of using the LUT method. Compared to the D5100 camera, the mean *E*_Ref_ using the RGBF and optimized RGBL cameras were reduced by 32.0% and 36.8%, respectively. Compared to the D5100 camera, the mean Δ*E*_00_ using the RGBF and optimized RGBS cameras were reduced by 5.3% and 60.5%, respectively. Compared to the RGBF camera, the advantage of using the optimized RGBL camera to obtain a smaller mean *E*_Ref_ was not significant; the advantage of using the optimized RGBS camera to obtain a smaller mean Δ*E*_00_ was significant. Compared to the RGBF camera, the mean Δ*E*_00_ using the optimized RGBS camera was reduced by 58.3%, but the mean *E*_Ref_ was increased by 28.6%. However, since the mean Δ*E*_00_ was as small as 0.3992, using the RGBF camera may be better than the optimized RGBS camera in spectral reflectance recovery due to the smaller mean *E*_Ref_ and no need to use the color filter applied to the fourth channel.

In this paragraph, the use of the wPCA method is discussed. Compared to the D5100 camera, the mean *E*_Ref_ values using the RGBF and optimized RGBL cameras were reduced by 21.6% and 28.2%, respectively. Compared to the D5100 camera, the mean Δ*E*_00_ values using the RGBF and optimized RGBS cameras were increased by 9.6% and reduced by 59.3%, respectively. Compared to the RGBF camera, the advantage of using the optimized RGBL camera to obtain smaller spectral error was also not significant. Compared to the RGBF camera, using the optimized RGBS camera had a 62.9% smaller mean Δ*E*_00_ but a 49.5% larger mean *E*_Ref_.

The RGBF camera is a compromised design for the case of using the LUT or wPCA method. If a mean *E*_Ref_ smaller than that of the RGBF camera is required, a suitable long-pass optical filter can be applied to the fourth channel. If an ultra-small color difference is required, a suitable short-pass optical filter can be applied to the fourth channel, but care must be taken not to add too much spectral error.

The computation time required for the LUT method is about two orders of magnitude faster than that required for reconstruction methods using basis spectra that emphasize the relationship between the test and training samples for the 3D case [27,29]. However, for the 4D case, the computation time required to use the LUT method is longer than the wPCA method, because it takes much longer time to locate the simplex for interpolation than the 3D case. For the case of using the quadcolor camera, the ratio of the computation time required to use the LUT method and wPCA method was 1:0.49, where samples were reconstructed from their signal vector *C* to the spectral reflectance vector ***S***_RefRec_ using MATLAB on the Windows 10 platform. The improvement of the algorithm to locate the simplex faster is a computational geometry problem and is beyond the scope of this paper.

## 5. Conclusions

Using the conventional tricolor cameras to recover the spectral reflectance has the advantages of low cost, high spatial resolution, fast detection, and no need to measure/estimate the camera spectral sensitivity functions. The reduction in the spectrum reconstruction error using the quadcolor cameras was shown, where the color filter array is compatible with the tricolor cameras. The wPCA and LUT methods were used to reconstruct spectra from the quadcolor camera signals. The optimized weighting factor of the wPCA method was used for individual cameras. The spectral error metrics, *E*_Ref_ and 1– GFC, and the color appearance error metrics, Δ*E*_00_ and SCI, were used to assess the reconstructed spectra. The assessment results for using the two spectrum reconstruction methods were compared.

It was assumed that the spectral sensitivities of the red, green and blue channels of the quadcolor cameras are the same as those of the Nikon D5100 camera. The spectral sensitivity of the fourth channel depends on the spectral transmittance of the IR-cut filter and the color filter in addition to the spectral sensitivity of the silicon sensor. The quadcolor RGBF camera is considered, where no color filter is applied to its fourth channel. The quadcolor RGBC, RGBS and RGBL cameras were also considered with sensitivity compensation filters, short-pass filters, and long-pass filters applied to their fourth channels, respectively. Five commercially available sensitivity compensation optical filters were used.

The Munsell color chips were taken as reflective surface examples where 202 and 1066 color chips were used to prepare reference/training and test samples, respectively, under the illuminant D65. It was found that using the LUT method, the number of outside samples that cannot be interpolated increases with the signal dimension. The outside samples were extrapolated using reference samples and the ARSs created with the CYMRGB color filters. The results are summarized below.

1. Comparison of the LUT and wPCA methods:

The advantage of using the LUT method compared to the wPCA method is that the mean spectral error is smaller and the spectral sensitivity functions of the camera are not required. Errors in the estimation of the camera spectral sensitivities can lead to additional errors in the reconstructed spectra using the wPCA method. This paper does not consider such errors. If included, the mean spectral error and color difference increase. The disadvantage of using the LUT method compared to the wPCA method is that the computation time is approximately doubled and the ARSs need to be measured for extrapolation. The computation time using the LUT method is longer because it takes time to locate the simplex for interpolation in the 4D signal space.

2. RGBF camera:

Compared to using the D5100 camera, using the RGBF camera effectively reduces the mean spectral error of the test samples even when no color filter was applied to the fourth channel. For the case of using the LUT method, the mean spectral error *E*_Ref_ = 0.0131 and 0.0089 using the D5100 and RGBF cameras, respectively. For the case of using the wPCA method, the mean spectral error *E*_Ref_ = 0.0121 and 0.0095 using the D5100 and RGBF cameras, respectively. Using the RGBF camera effectively reduces the mean spectral error compared to the D5100 camera when the LUT method or wPCA method is used to reconstruct spectra.

3. RGBL camera:

A long-pass optical filter can be applied to the fourth channel for further reducing the mean spectral error. Using the optical filter optimized for the minimum mean spectral error metric *E*_Ref_, the mean *E*_Ref_ = 0.0083 and 0.0087 for the cases of using the LUT and wPCA methods, respectively. The reduction in the mean spectral error is not significant using the optimized RGBL camera compared to using the RGBF camera.

4. RGBS camera:

A short-pass optical filter can be applied to the fourth channel for reducing the mean color difference of the test samples, but the mean spectral error is larger. Using the optical filter optimized for the mean minimum mean color difference Δ*E*_00_, the mean Δ*E*_00_ = 0.1666 and 0.158 for the cases of using the LUT and wPCA methods, respectively; the maximum Δ*E*_00_ = 1.0169 and 4.1594 for the cases of using the LUT and wPCA methods, respectively. The mean and maximum Δ*E*_00_ using the LUT method are small, so the optimized RGBS camera using the LUT method may be suitable as an imaging colorimeter.

5. Zero-color-difference condition:

The zero-color-difference conditions using the LUT method and the wPCA method were given, respectively. If the camera sensitivity functions ideally fit the CIE CMFs, then the color difference between the reflection light spectrum and the spectrum reconstructed using the LUT method is zero. For the case of using the wPCA method, the color difference condition is also zero if the additional condition is satisfied, which requires that the spectral amplitude calculated from Equation (5) be non-negative.

6. Color matching function mismatching factor (CMFMisF):

From the zero-color-difference condition, CMFMisF is defined to represent the RMS error of fitting the CMFs using the spectral sensitivity functions of a camera. An empirical design rule to keep the color difference small is to reduce the value of CMFMisF.

Since the red, green and blue channels of the considered quadcolor cameras were assumed to be the RGB channels of the D5100 camera, it is possible to design color filters for all four channels for further improving the spectral reflectance recovery. Future developments are shown below.

1. The relation of the color difference and camera spectral sensitivity functions was shown in this paper. More research is needed to investigate the relation of the spectral error and camera spectral sensitivity functions.
2. Methods are needed to optimize the camera spectral sensitivity functions to achieve low spectral error and low color difference.
3. Time-saving algorithms need to be developed for the LUT method in the 4D case.

## Figures and Tables

**Table 1:** Assessment metric statistics for the spectrum reconstruction of test samples using the D5100 and RGBF cameras. The spectrum reconstruction method is the LUT method.

Metric	Camera	Nikon D5100			RGBF		
	Sample	All	Inside	Outside	All	Inside	Outside
	No.	1066	864	202	1066	726	340
*E*_Ref_	mean *μ*	0.0131	0.0120	0.0180	0.0089	0.0077	0.0115
	std *σ*	0.0124	0.0107	0.0169	0.0079	0.0064	0.0100
	*PC50*	0.0089	0.0087	0.0125	0.0064	0.0055	0.0082
	*PC98*	0.0540	0.0485	0.0698	0.0345	0.0262	0.0416
	*MAX*	0.1038	0.0859	0.1038	0.0567	0.0451	0.0567
*GFC*	mean *μ*	0.9971	0.9974	0.9961	0.9989	0.9993	0.9980
	std *σ*	0.0074	0.0071	0.0085	0.0023	0.0012	0.0035
	PC50	0.9993	0.9994	0.9985	0.9996	0.9997	0.9992
	*MIN*	0.9000	0.9000	0.9325	0.9669	0.9832	0.9669
	*RGF99*	0.9343	0.9375	0.9208	0.9887	0.9972	0.9706
Δ*E*_00_	mean *μ*	0.4215	0.4239	0.4111	0.3992	0.3865	0.4263
	std *σ*	0.4065	0.4182	0.3529	0.3590	0.3535	0.3696
	*PC50*	0.2796	0.2795	0.2830	0.2792	0.2734	0.2902
	*PC98*	1.6478	1.6900	1.4386	1.4447	1.4441	1.4453
	*MAX*	2.5918	2.5918	1.8207	1.8843	1.8843	1.8099
*SCI*	mean *μ*	4.1253	3.7503	5.7291	3.5296	2.9027	4.8682
	std *σ*	3.2381	2.9266	3.9487	2.7643	2.0677	3.4962
	*PC50*	3.1484	2.9310	4.7827	2.7585	2.3611	3.9021
	*PC98*	13.7172	12.1239	16.4027	11.9210	9.1115	16.2671
	*MAX*	25.3596	25.2299	25.3596	20.0506	14.5365	20.0506

**Table 2:** The same as Table 1, except that the spectrum reconstruction method is the wPCA method. The optimized *γ* = 1.7 and 1.2 using the D5100 and RGBF cameras, respectively.

Metric	Method	Nikon D5100			RGBF		
	Sample	All	Inside	Outside	All	Inside	Outside
	No.	1066	864	202	1066	726	340
*E*_Ref_	mean *μ*	0.0121	0.0110	0.0169	0.0095	0.0080	0.0127
	std *σ*	0.0121	0.0098	0.0181	0.0087	0.0068	0.0110
	*PC50*	0.0086	0.0082	0.0115	0.0066	0.0059	0.0091
	*PC98*	0.0531	0.0437	0.0783	0.0338	0.0288	0.0490
	*MAX*	0.1152	0.0817	0.1152	0.0742	0.0742	0.0738
*GFC*	mean *μ*	0.9978	0.9984	0.9950	0.9985	0.9992	0.9972
	std *σ*	0.0056	0.0032	0.0108	0.0046	0.0029	0.0068
	PC50	0.9994	0.9994	0.9988	0.9995	0.9996	0.9990
	*MIN*	0.9017	0.9618	0.9017	0.9315	0.9364	0.9315
	*RGF99*	0.9493	0.9676	0.8713	0.9765	0.9945	0.9382
Δ*E*_00_	mean *μ*	0.3884	0.3443	0.5769	0.4257	0.3475	0.5926
	std *σ*	0.4133	0.3235	0.6416	0.4558	0.3213	0.6252
	*PC50*	0.2573	0.2422	0.3259	0.2942	0.2689	0.3985
	*PC98*	1.8615	1.3261	2.5734	1.7690	1.1557	2.6548
	*MAX*	2.8330	2.5378	2.8330	5.0328	3.3353	5.0328
*SCI*	mean *μ*	3.8000	3.1831	6.4384	3.8938	2.9955	5.8118
	std *σ*	3.9295	2.7777	6.3288	4.2229	2.9221	5.6872
	*PC50*	2.6841	2.5232	4.3897	2.6196	2.2980	4.0662
	*PC98*	16.7371	11.2917	29.0811	15.9216	9.1608	19.4698
	*MAX*	34.6758	28.8125	34.6758	46.2716	42.5477	46.2716

**Table 3:** The values of *E*_Ref_ for the cases shown in Figure 9a–f and Figure 10a–f. The values larger than 0.03 are shown in bold.

Method	LUT/Figure 9a–f					wPCA/Figure 10a–f				
Camera	D5100	RGBF	RGBC	RGBS	RGBL	D5100	RGBF	RGBC	RGBS	RGBL
(a)	0.0040	0.0032	0.0033	0.0048	0.0033	0.0093	0.0124	0.0123	0.0059	0.0106
(b)	0.0220	0.0108	0.0108	0.0230	0.0135	0.0157	0.0076	0.0076	0.0124	0.0069
(c)	0.0129	0.0071	0.0190	0.0139	0.0063	**0.0462**	0.0076	0.0112	0.0178	0.0085
(d)	0.0244	0.0229	0.0229	0.0252	0.0227	0.0221	0.0260	0.0242	0.0063	0.0227
(e)	0.0081	0.0082	0.0109	0.0152	0.0084	0.0181	0.0203	0.0190	0.0068	0.0177
(f)	0.0242	**0.0308**	0.0292	0.0275	0.0252	**0.0392**	**0.0343**	**0.0343**	**0.4207**	**0.0341**

**Table 4:** The values of Δ*E*_00_ for the cases shown in Figure 9a–f and Figure 10a–f. The values larger than 1.0 are shown in bold.

Method	LUT/Figure 9a–f					wPCA/Figure 10a–f				
Camera	D5100	RGBF	RGBC	RGBS	RGBL	D5100	RGBF	RGBC	RGBS	RGBL
(a)	0.1065	0.1043	0.1056	0.1283	0.1028	0.1986	0.5428	0.5415	0.1529	0.4654
(b)	0.1092	0.1395	0.1398	0.1755	0.1836	0.0290	0.1045	0.1053	0.0422	0.0879
(c)	0.0934	0.0852	0.2068	0.0663	0.0507	0.4602	0.1721	0.3605	0.2063	0.2128
(d)	0.6975	0.6439	0.6442	0.1811	0.6077	0.6969	0.8374	0.7762	0.0611	0.7220
(e)	0.7396	0.7238	0.9626	0.4721	0.6973	**1.8552**	**2.0505**	**1.8888**	0.3183	**1.7703**
(f)	0.4089	0.7133	0.6205	0.2830	0.5285	**1.0557**	**1.1166**	**1.0400**	**4.1594**	**1.1133**

**Table 5:** Assessment metric statistics for the spectrum reconstruction of the 1066 test samples. The quadcolor cameras and spectrum reconstruction methods are indicated. For the case of using the wPCA method, the optimized *γ* = 1.2, 1.2, 1.9 and 1.3 using the RGBF, RGBC, RGBS and RGBL cameras, respectively. The best values are shown in bold.

Metric	Method	LUT				wPCA			
	Camera	RGBF	RGBC	RGBS	RGBL	RGBF	RGBC	RGBS	RGBL
*E*_Ref_	mean μ	0.0089	0.0091	0.0115	**0.0083**	0.0095	0.0098	0.0141	0.0087
	std σ	0.0079	0.0082	0.0135	0.0072	0.0087	0.0103	0.0328	0.0081
	PC50	0.0064	0.0064	0.0074	0.0061	0.0066	0.0070	**0.0058**	0.0061
	PC98	0.0345	0.0348	0.0579	**0.0310**	0.0338	0.0353	0.0944	0.0317
	MAX	0.0567	0.0623	0.1244	**0.0490**	0.0742	0.1722	0.4207	0.0975
*GFC*	mean μ	0.9989	0.9988	0.9978	**0.9990**	0.9985	0.9984	0.9925	0.9988
	std σ	0.0023	0.0025	0.0066	0.0023	0.0046	0.0070	0.0415	0.0033
	PC50	0.9996	0.9996	0.9996	**0.9997**	0.9995	0.9995	**0.9997**	0.9996
	MIN	**0.9669**	0.9659	0.9128	0.9594	0.9315	0.8197	0.3628	0.9417
	RGF99	0.9887	0.9897	0.9512	**0.9897**	0.9765	0.9747	0.9212	0.9812
Δ*E*_00_	mean μ	0.3992	0.3987	0.1666	0.3507	0.4257	0.4434	**0.1580**	0.3862
	std σ	0.3590	0.3596	0.1298	0.3297	0.4558	0.6440	0.2640	0.4745
	PC50	0.2792	0.2871	0.1313	0.2409	0.2942	0.3071	**0.0971**	0.2610
	PC98	1.4447	1.4866	**0.5588**	1.3806	1.7690	1.8975	0.7307	1.7779
	MAX	1.8843	1.7817	**1.0169**	2.1648	5.0328	15.6619	4.1594	8.1484
*SCI*	mean μ	3.5296	3.5722	3.0033	3.2529	3.8938	4.0839	**2.9014**	3.4897
	std σ	2.7643	2.7926	2.7587	2.5591	4.2229	5.8323	4.8805	3.6736
	PC50	2.7585	2.7202	2.2204	2.5913	2.6196	2.6887	**1.6923**	2.3663
	PC98	11.9210	12.2766	11.6279	**10.8224**	15.9216	16.0729	14.5862	15.7454
	MAX	20.0506	**18.8735**	30.1699	20.8300	46.2716	129.481	75.332	36.3269

## Data Availability

(1) Spectral sensitivities of the Nikon D5100 camera are available: http://spectralestimation.wordpress.com/data/. (2) Spectral reflectance of matt Munsell color chips are available: https://sites.uef.fi/spectral/munsell-colors-matt-spectrofotometer-measured/. (3) Spectral transmittance of the UV/IR cut filter is available: https://agenaastro.com/downloads/manuals/baader-uvir-cut-filter-stat-sheet.pdf. (4) Spectral transmittance of the sensitivity compensation color filters are available: https://www.isuzuglass.com/products/glass-iec.html. All are accessed on 12 July 2022.

## Abbreviations

ARS	Auxiliary Reference Sample
CFA	Color Filter Array
CMF	Color-Matching Function
CMFMisF	Color-Matching Function Mismatch Factor
CMY	Cyan, Magenta and Yellow
CMYRGB	Cyan, Magenta, Yellow, Red, Green and Blue
GFC	Goodness-of-Fit Coefficient
LUT	Look-Up Table
MAX	Maximum
MIN	Minimum
NMT	Non-Negative Matrix Transformation
PC50	50th Percentile
PC98	98th Percentile
PCA	Principal Component Analysis
RGB	Red, Green and Blue
RGF99	Ratio of Good Fit (the ratio of samples with *GFC* > 0.99)
RMS	Root Mean Square
RPRM	Root Polynomial Regression Model
SCI	Spectral Comparison Index
wPCA	weighted Principal Component Analysis

## Appendix A. Zero-Color-Difference Condition Using the LUT Method

The CMF matrix is defined as ***F***= [***u****_x_**u**_y_**u**_z_*], where ***u****_x_*, ***u****_y_*, and ***u****_z_* are the CMF vectors defined in Section 3.4. If the camera spectral sensitivity functions fit the CMFs ideally, the CMF matrix ***F*** can be written as

***F*** = ***D***_Cam_***B***^T^, (A1)

where ***D***_Cam_ is the camera spectral sensitivity matrix defined in Section 3.1; ***B*** is a 3x4 coefficient matrix. If Equation (A1) is valid, the color difference between the reflection light spectrum ***S***_Reflection_ and the spectrum ***S***_Rec_ reconstructed using the LUT method is zero.

Proof: From Equation (A1), the tristimulus vectors of the reflection light spectrum and the *i*-th reference spectrum ***S****_i_* can be written as

***T***_Reflection_ = ***F***^T^***S***_Reflection_ (A2)

and

***T***_i_ = ***F***^T^***S***_i_, (A3)

respectively. In Equation (6), the signal and *i*-th reference vectors are

***C*** = ***D***_Cam_^T^***S***_Reflection_, (A4)

and

***C****_i_* = ***D***_Cam_^T^***S****_i_*, (A5)

respectively. Substituting Equations (A4) and (A5) into Equation (6) and multiplying both sides of Equation (6) with the coefficient matrix ***B***, we have

(A6) $${{\mathrm{BD}}_{\mathrm{Cam}}}^{T}S_{\mathrm{Reflection}}=\sum_{i=1}^{5}{{\alpha_{i}}_{\mathrm{Cam}}}^{T}S_{i}.$$

From Equations (A1) and (A6),

(A7) $$F^{T}S_{\mathrm{Reflection}}=\sum_{i=1}^{5}{\alpha_{i}}^{T}S_{i}.$$

Substituting Equations (A2) and (A3) into Equation (A7), we have

(A8) $${T_{\mathrm{Reflection}}}^{\;}=\sum_{i=1}^{5}\alpha_{i}.$$

Multiplying both sides of Equation (8) with the transpose of the CMF matrix ***F***, we have

(A9) $$F^{T}S_{\mathrm{Rec}}=\sum_{i=1}^{5}{\alpha_{i}}^{T}S_{i}.$$

Substituting Equations (A2) and (A3) into Equation (A9), we have

(A10) $${T_{\mathrm{Rec}}}^{\;}=\sum_{i=1}^{5}\alpha_{i}.$$

From Equations (A8) and (A10), ***T***_Rec_ = ***T***_Reflection_. Therefore, the color difference between the reflection light spectrum and the reconstructed spectrum is zero. Since no assumptions are made about the reflection light spectrum, the result of the zero color difference is general. □

## Appendix B. Zero-Color-Difference Condition Using the wPCA Method

If Equation (A1) is valid and the spectral amplitude calculated from Equation (5) is non-negative, then the color difference between the reflection light spectrum ***S***_Reflection_ and the spectrum ***S***_Rec_ reconstructed using the wPCA method is zero. Following a similar procedure in Appendix A, from Equation (5), we have

(A11) $${T_{\mathrm{Reflection}}}^{\;}={T_{\mathrm{Rec}}}^{\;}=T0_{\;}+\sum_{k=1}^{4}d_{k},$$

where ***T***_0_ = ***F***^T^***P***_0_ and ***T****_k_* = ***F***^T^***P***_k_. Therefore, the color difference between ***S***_Reflection_ and ***S***_Rec_ is zero.

However, the negative spectral amplitude calculated from Equation (5) is not uncommon due to the truncated higher-order principal components in Equation (5). In practice, the color difference between the reflection light spectrum and the spectrum reconstructed using the wPCA method is usually non-zero, even if Equation (A1) is valid.
